# Supplementary figures and images for: A Geometric Theory Integrating Human Binocular Vision With Eye Movement
Source: Front Neurosci. 2020 Dec 7;14:555965. doi: 10.3389/fnins.2020.555965 (PMC7750472; doi:10.3389/fnins.2020.555965)

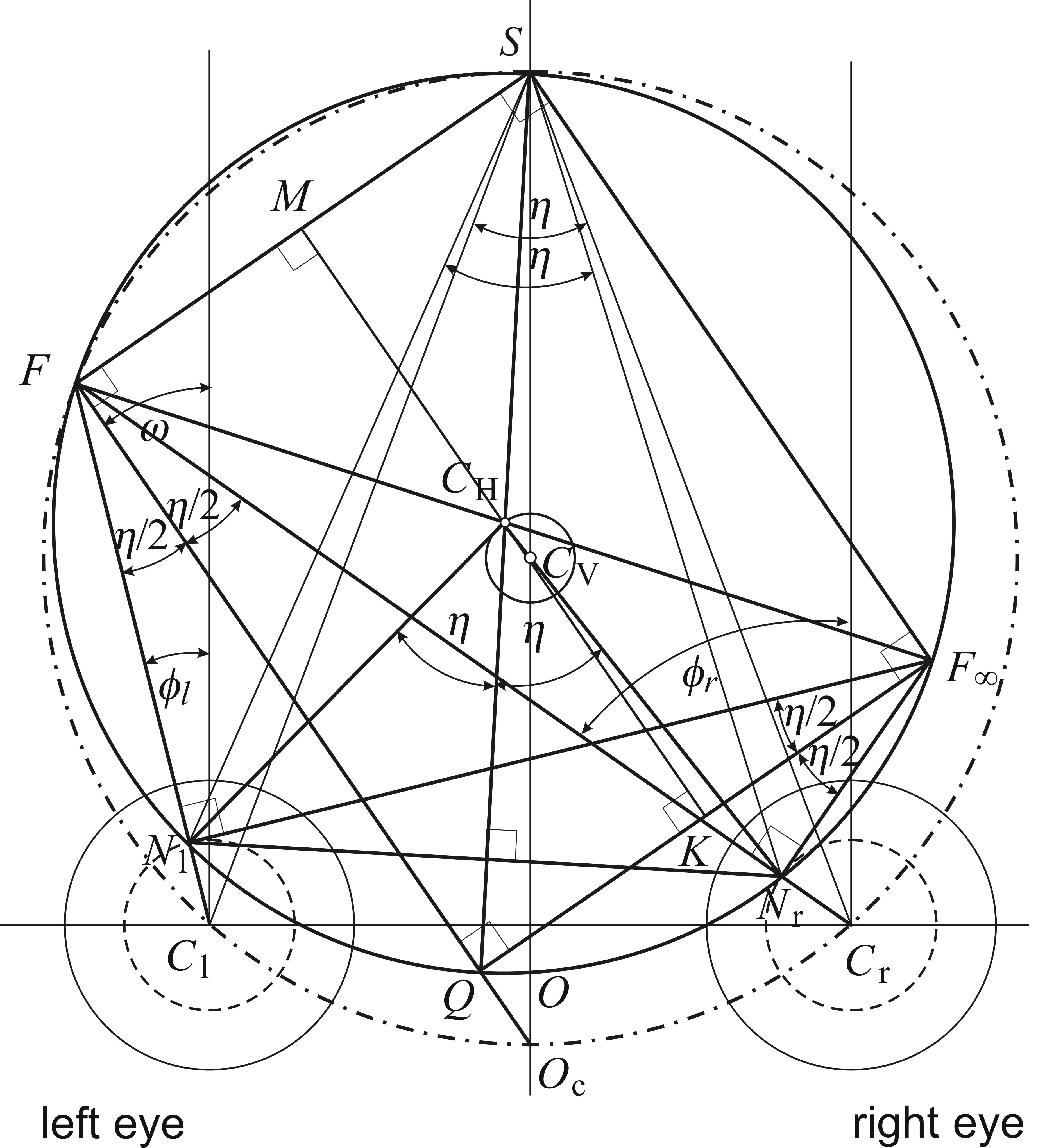

Supplement: Supplementary file 1 [file Image_1.JPEG]
