# Supplementary material for: A Geometric Theory Integrating Human Binocular Vision With Eye Movement
Source: Front Neurosci. 2020 Dec 7;14:555965. doi: 10.3389/fnins.2020.555965 (PMC7750472; doi:10.3389/fnins.2020.555965)
Supplement: Supplementary file 2 [file Data_Sheet_2.pdf]

Supplementary Material

SUPPLEMENT 1

The proof of Proposition 1 uses results obtained in (Turski, 2016), which are shown in Figure S1 below. The large circle drawn in a dash-dot line is the Vieth-Müller circle (VMC) with its center C_V and the large circle drawn with a solid line is the geometric horopter circle (GHC) with center C_H. The vergence is $\eta = \phi_r - \phi_l$ and the version is $\omega = 1/2(\phi_r + \phi_l)$. The point S of symmetric convergence is the intersection of the VMC with the GHC.

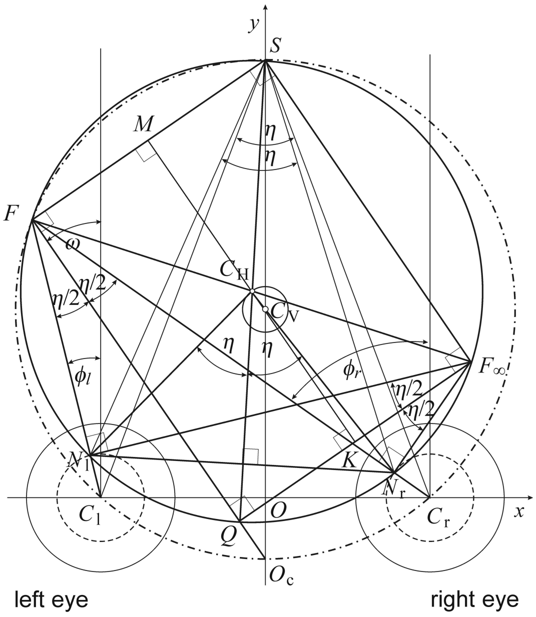

Figure S1. Proof of Proposition 1

PROPOSITION 1. *Let the nodal point be located on the optical axis at any point at or between the spherical eyeball's rotation center and its pupil. Then, for the binocular eyes position with fixation point F in the horizontal visual plane, the lines passing through the nodal points and perpendicular to the visual axes intersect at the point F_∞ on the circular horopter. It then follows that line segment FF_∞ must pass through the horopteric circle's center.*

PROOF. When the eyes are fixated on F, the GHC is defined by three points: F and the two nodal points, N_r and N_l. According to the Central Angle Theorem, the rays perpendicular to the visual axes at the nodal points intersect at point F_∞ on the GHC. This is demonstrated by the triangles $\triangle QN_lF$ and $\triangle QN_lF_\infty$ in Figure S1 sharing the same angle at vertices F andF_∞. Then, by results proved in (Turski, 2016), line segment FF_∞'s midpoint C_H is the center of inscribed rectangle $\square QF_\infty SF$ and, therefore, also the center of GHC's center. This completes the proof. □

SUPPLEMENT 2

A. Binocular Conics Transformation

The *GeoGebra* applet BCT at https://www.geogebra.org/m/ncssdqsd allows us to visualize the binocular conics' (shown in red) transformations in the visual plane of bifoveal fixations. The initial abathic-distance fixation F has coordinates $(0, 99.61)$. The initial conic at this resting vergence position consists of two parallel horizontal lines where the line passing through F is the linear horopter. When point F moves, the window ResetF displays the point F's current coordinates. After the session is finished, return F to the position on the intersection of the y-axis and the AIS circle that locks to $(0, 100)$, so reset coordinates in ResetF from $(0, 100)$ to $(0, 99.61)$ to return F to the resting vergence position.

1. Open the above link in your browser.
2. In the applet, click on the red F of the resting vergence position at $(0, 99.61)$ to highligtht the red dot.
3. Drag the red point with your mouse through the visual plane to transform the conics.
4. Return F to the resting vergence position (described above) by resetting coordinates in ResetF to $(0, 99.61)$.

B. Retinal Correspondence

Remark 1 in Section 5, asserts that the retinal correspondence defined in RETINAL CORRESPONDENCE in Section 3 is well defined concept. I demonstrate here this assertion with *GeoGebra*'s simulation of binocular conics transformation. In the*GeoGebra* applet BCT, the point Q on the linear horopter (shown in blue) is projected along visual lines to points Q_r and Q_l on the right and left image planes, respectively. The angles between these visual lines of point Q and the corresponding initial visual axes of F are displayed as ϕ_{Qr} and ϕ_{Ql} for the right and left eye respectively. When the resting vergence position fixation point F is moved (cf. the previous subsection), the value of these angles unchanged to 3 decimal places. This proves that the retinal correspondence relationship defined in Section 3 of the article is invariant of eyes position in the visual plane of bifoveal fixations and, therefore, is a well-defined concept.

REFERENCES

Turski, J. (2016). On binocular vision: The geometric horopter and cyclopean eye. *Vision Research* 119, 73–81. doi:10.1016/j.visres.2015.11.001
